# The utilisation of domestic goats in rural and peri-urban areas of KwaZulu-Natal, South Africa

**DOI:** 10.1007/s11250-023-03587-3

**Published:** 2023-05-17

**Authors:** Anele Aurelia Khowa, Zivanai Tsvuura, Rob Slotow, Manqhai Kraai

**Affiliations:** 1 Centre for Functional Biodiversity, School of Life Sciences, University of KwaZulu-Natal, Private Bag X01, Scottsville, 3209 South Africa; 2 Department of Biological and Agricultural Sciences, School of Natural and Applied Sciences, Sol Plaatje University, P. Bag X5008, Kimberley, 8300 South Africa

**Keywords:** Consumption, Homesteads, Livelihoods, Livestock, Sales of goats

## Abstract

Goats are a common ruminant in livestock husbandry systems in communal areas of southern Africa, but less so in peri-urban areas. While dynamics of goat farming in the former are relatively well-understood, little is known about it in peri-urban environments. We investigated the contribution of small-scale farming of goats to household livelihoods in rural and peri-urban areas in KwaZulu-Natal Province of South Africa. We used a semi-structured questionnaire survey to elicit views of 115 respondents on the contribution of goats to household income at two rural (Kokstad, Msinga) and two peri-urban (Howick, Pietermaritzburg) locations. Goats contributed to household income as a source of cash and meat in various sociocultural contexts such as at weddings, funerals and festive period gatherings (e.g. Easter and Christmas), including payment for household needs and expenses including food, school fees and medico-cultural consultations. These findings were more pronounced in rural areas, where more goats were kept than in peri-urban areas, which also had smaller herds per household. Goats were a source of cash in numerous ways including sales of their skins after slaughter and use of skins through value addition to make household craft items such as stools that could be sold for cash. None of the farmers milked their goats. Goat famers also kept cattle (52%), sheep (23%) and chickens (67%). Goat ownership appeared to be more profitable in rural areas and contributed to a lesser extent as a source of income in peri-urban areas where goats were largely kept for sale. There is potential for increased value addition of goat products to increase returns from small-scale goat farming in rural and peri-urban settings. Artefacts and cultural symbols derived from goat products are pervasive amongst Zulu people, and represent an additional avenue of research into ‘hidden’ valuation of goats.

**Supplementary information:**

The online version contains supplementary material available at 10.1007/s11250-023-03587-3.

## Introduction

Proximity to resources, education and opportunity has, for decades, resulted in the move of people from rural to peri-urban areas of southern Africa (Greiner 2011; Shackleton et al. 2013). Peri-urban areas, a feature of all urban areas throughout the world, are characterised by land use types different to either urban or rural (Mbiba and Huchzermeyer 2002; Mortoja et al. 2020). The move closer to the major towns has become permanent for most, which left land in rural homesteads abandoned, while peri-urban areas became crowded with original inhabitants and those drawn by the nearby towns (see Shackleton et al., 2013). There is therefore increased competition for land close to cities, which results in small space allocations to residents (Kidido and Bugri 2020). Limited space in peri-urban areas may further reduce land available to herbivorous livestock (Omiti et al. 2009). This may, in turn, have negative implications for animal production and livelihoods of their owners for whom keeping livestock is part of their traditional practices (Kitole and Sesabo 2022). Specifically, peri-urban areas have limited space for foraging livestock, which may affect their ability to meet their energy requirements (Galhena et al. 2013), and also compromise food security, livelihoods and cultural uses of livestock, and may negatively impact social cohesion.

Alternatives for meeting human livelihoods and well-being are important due to increasing demands for food (Tito et al. 2018; Mbow et al. 2019). As such, there are greater socio-economic and socio-environmental constraints to food production, distribution and access in sub-Saharan Africa and other parts of the developing world (Rust and Rust 2013). Such constraints may be severe for smallholder farmers in rural areas. Social status and gender may influence the ability to obtain food (Baiphethi and Jacobs 2009; Dean and Sharkey 2011). Food access challenges may be more pronounced in rural and peri-urban areas than urban areas (Dean and Sharkey 2011; Motbainor et al. 2016). Peri-urban areas are also characterised by people moving from rural areas in search of better opportunities like employment, education and healthcare or urban dwellers who are looking to acquire larger plots of land at lower prices than would be available in the cities (Simon 2008). The cost of living in peri-urban areas is higher than rural areas, where expenses like water, rent and electricity are low (Chen and Ravallion 2007; Tawodzera 2012), and exempted from municipal rates (Baiphethi and Jacobs 2009). Despite this, people in rural areas are generally considered poor (Baiphethi and Jacobs 2009), and rely on natural resources to earn a living (Lebbie 2004), such as rangelands for small-scale animal and crop farming (Grace et al. 2017).

 Small-scale or subsistence farming of livestock such as goats, sheep, cattle and chickens is associated with rural and peri-urban communities. Small-scale and subsistence farming may contribute to household food and nutrition security, and act as a source of income for families (Aliber and Hart 2009; Baiphethi and Jacobs 2009), but does not contribute significantly to the major meat markets of a country (Aliber and Hart 2009; Ngomane et al. 2022, but see Hoag 2018). In addition, rural and peri-urban households may be sustained through government social welfare grants, such as in South Africa (Labadarios et al. 2011; D'Haese et al. 2013) and Namibia (Greiner 2011).

Small-scale livestock farmers produce animal products at low input cost to households by relying on natural, communally owned rangeland for animal feed, which in turn results in greater financial returns when sales are made (Lebbie 2004). Animals that are relatively inexpensive and versatile to keep for small-scale farmers are preferred, and goats meet these criteria (Lebbie 2004; Peacock 2005; Nair et al. 2021). In addition, small-scale farmers favour goats due to their mixed feeding abilities, high reproductive potential, early maturity and ability to survive without supplemental feeds in resource poor environments (Peacock, 2005; Peacock and Sherman 2010). Moreover, goats are resistant to diseases and so have low veterinary input costs (Nair et al. 2021). One reason contributing to goats’ good performance without supplementation may be that the rural areas have considerable amounts of natural vegetation that livestock can feed on, which sustains them even during times of drought (Reeder and Kramer 2005; Uzun 2005). However, livestock farming in more arid environments may be associated with greater requirements for supplementation of forage (Müller et al. 2015; Charambira et al. 2021).

Goats are tolerant to droughts, diseases and pests (Darcan and Silanikove 2018; Nair et al. 2021). As such, the range within which goats occur is extensive. For example, goats occur in all agroecological regions of South Africa (Mogala 2012), which range from humid through mesic to semi-arid and arid regions. Another reason for goat suitability to small-scale farmers is their quick recovery and high reproductive rates, which allow herds to increase in numbers quickly after a drought (Fatet et al. 2011; Nair et al. 2021). Specifically, goats have inter-kidding intervals of less than a year (0.75 years), while cattle and sheep have inter-calving intervals of more than a year (Hare et al. 2006). Moreover, the reproductive cycle of goats is also not limited by season (Fatet et al. 2011; Kraai et al. 2022). Goats are, thus, popular for small-scale farmers compared to other livestock such as cattle. Little is known about non-commercial livestock farming in peri-urban areas. Also, information is lacking on the type of domestic animals preferred in peri-urban areas. Furthermore, the contribution of goats to livelihoods and uses of their products at household levels are unknown, more so because a large number of the animals are sold at informal markets (Lebbie 2004).

Although goat farming is considered favourable in small-scale farming systems in resource-poor environments, the negative environmental impacts of the animal are widely acknowledged (e.g. Lipson et al., 2019; Monau et al. 2020). In particular, the feeding and trampling activities of goats are associated with biodiversity loss, increased run-off and soil erosion (Khan et al. 2015; Menezes et al. 2021). The study aimed to determine the role of goats to household livelihoods in rural and peri-urban areas of KwaZulu-Natal in South Africa. The objectives were to (1) quantify the use of goats by households for meat, milk and generating income; (2) identify and quantify additional uses of goats or their products (e.g. manure, skins) as part of a localised goat value chain; (3) determine other household income streams; and (4) determine the frequency with which goat farmers participate in near to distant formal livestock markets. Our assumptions were that (1) there would be greater herd sizes, more livestock and increased use of goats for meat and generating household income in rural compared to peri-urban areas; (2) there would be greater use of secondary products of goats in rural than in peri-urban areas; and lastly, (3) peri-urban areas will have an increased reliance on other household income alternatives than rural areas.

## Materials and methods

### Study sites

The study was carried out at several rural and peri-urban sites located near the towns of Howick, Kokstad, Pietermaritzburg and Tugela Ferry, all in KwaZulu-Natal Province of South Africa. The biophysical features of all the study sites are described in Table S1. Pietermaritzburg, Howick and Kokstad occur in *Themeda triandra* grasslands that have been transformed by overutilisation so that *Aristida junciformis* now dominates at several places (Mucina and Rutherford 2006). The geology of Pietermaritzburg, Howick and Kokstad is dominated by apedal and plinthic soil forms, which, together with the mean annual precipitation of > 740 mm and temperate to moderate subtropical summer temperatures, make the areas suitable for dry land crop production (Mucina and Rutherford 2006). Msinga, where Tugela Ferry is located, is a rural area with greater tree cover than the other sites. It is characterised as semi-arid to mesic (ca. 650 mm mean annual precipitation) savanna whose vegetation consists of short (1.5–10 m) deciduous broad-leaved trees and a sparse herbaceous layer of forbs and grasses (Nzimande et al. 2022). Soils are shallow, reddish-brown gritty sands with low amounts of clay and organic matter, which, coupled with relatively lower rainfall and high summer temperatures, limits crop production.

### Sampling

Data collection took place between May and December 2020, using a structured questionnaire survey. We conducted questionnaire surveys with farmers from different households selected using the snowball sampling technique (Atkinson and Flint 2001). Questions relating to the sale of goats, frequency of their participation at distant markets, and the prices at which the goats sold were asked to determine the amount of money farmers obtained through sales. Once this was determined, we went further to ask how the monies were spent. To determine whether farmers had other sources of income that contribute to meeting household needs, we asked questions on other livelihood activities such as crop production or keeping of other livestock (e.g. cattle, sheep and chickens), as well as quantify other income sources such as social grants. The questionnaire also included questions that aimed to assess people’s socio-economic dynamics and demography (e.g. age, sex, and educational attainment, employment status) to the level at which livestock contributed to household needs. Questions regarding uses of other animal products were included.

Household questionnaire surveys were carried out at each homestead. The questionnaires were originally prepared in isiZulu and English. The questions were then asked in the recipients’ preferred language if the respondent could not read as per the questionnaire handed out to them. However, those who were literate read and responded to the questions themselves without any need for reading the questions to them. Most of the respondents were of the Zulu ethnic group, which meant no interpreter was required as the researcher (AAK) is fluent in the isiZulu language. The initial target for the survey questions at each homestead was the head of the household, who is expected to be well informed about aspects of the household pertaining to livestock and how it is used. In some instances, where ownership of a household’s goat herd was shared amongst the family, questions were answered in a collaborative effort with only the main owner or elder’s demographic details in the household being taken.

### Data analysis

The questionnaire survey data were managed and analysed using inferential and descriptive statistics. In inferential statistics, Pearson’s chi-square test was performed with the area (rural/peri-urban) as the independent variable and price of adult and subadult goats, the purpose of keeping goats, source of income and the level of education of the farmers as the dependent variables. Specifically, we compared the cost of goats between rural and peri-urban areas for adult and subadult goats. Chi-square analysis was also used to determine the main sources of income between households in rural and peri-urban areas. An independent samples *t*-test was used to compare herd sizes of homesteads in rural and peri-urban areas. Descriptive statistics were run to determine means, frequencies and percentages of the social and economic characteristics of the farming practices in rural and peri-urban areas.

## Results

A total of 115 respondents were interviewed from the four study sites. Rural areas accounted for 55 respondents, while 60 were from peri-urban areas. There were 36 female and 19 male respondents in the rural areas. Peri-urban areas had 23 female and 37 male respondents. Overall, 51% of respondents were female. Fifty-one percent of the respondents were the elderly (between 50 and 70 years), with the lowest (11%) representation coming from the young adults (25–34 years). The majority (66%) of households had a male head. Overall, there was an employment rate of 39% across rural and peri-urban areas, with 17% rural employment and 22% peri-urban employment. Most (57%) of the respondents had little to no formal education. The level of education was similar between male and female respondents (*χ*^2^ = 10.218, df = 5, *P* = 0.069). None of the female respondents had tertiary level education, while slightly more males attained primary level education (Table S2).

The aim of keeping goats was similar (*χ*^2^ = 5.833, df = 3, *P* = 0.212) between rural and peri-urban areas. Respondents in both rural and peri-urban areas primarily owned goats for a combination of subsistence use and sales (Table 1). Urgent sales of goats were high in both rural (82%) and peri-urban areas (73%). More rural farmers (66%) indicated slaughtering goats for household meat consumption than peri-urban (48%) goat farmers. Ownership for traditional uses was also important in both areas but tended to be greater in peri-urban than in rural areas. Most of the goat farmers also kept other livestock which were dominated by cattle, sheep and chickens. Some 53% of goat farmers in rural areas also farmed cattle, which is considerably greater than the 35% for peri-urban goat farmers (Fig. S1). Goat farmers in peri-urban areas farmed sheep infrequently (7%), which increased to 40% in rural areas. For chickens, there were equal proportions (67%) of goat famers keeping them in rural and peri-urban areas (Fig. S1).

**Table 1:** The aim of keeping goats by farmers in rural and peri-urban areas in KwaZulu-Natal, South Africa

Purpose of goat ownership				Total (%)
		Rural (%)	Peri-urban (%)	
	Subsistence use	2	3	5
	Sales	5	4	9
	Subsistence use and sales	33	27	60
	Traditional ceremonies	8	17	25
Total		48	52	100

**Fig. 1:**
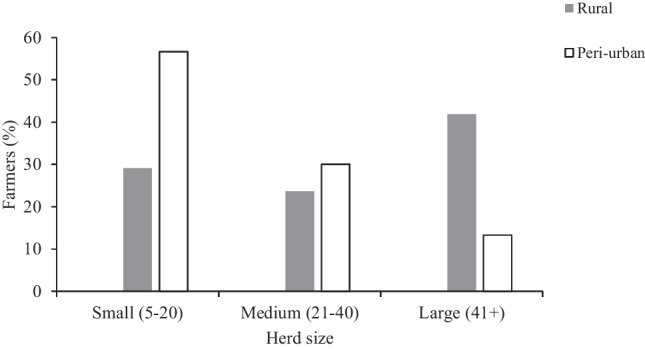
Herd sizes of goats in rural and peri-urban area homesteads in KwaZulu-Natal, South Africa

Herd sizes of goats differed significantly between rural and peri-urban areas (*t* = 2.038, df = 114, *P* = 0.044). Peri-urban areas had the highest number of small herds of goats (57%), while the majority (42%) of large herds were in rural areas (Fig. 1). Sizes of the herds of other livestock types (e.g. cattle, sheep) also differed between rural and peri-urban areas (Fig. S1). In particular, medium to large herds (> 20 animals) of sheep were absent in peri-urban areas. Overall, goats were the most dominant livestock occurring in all herd size ranges.

**Fig. 2:**
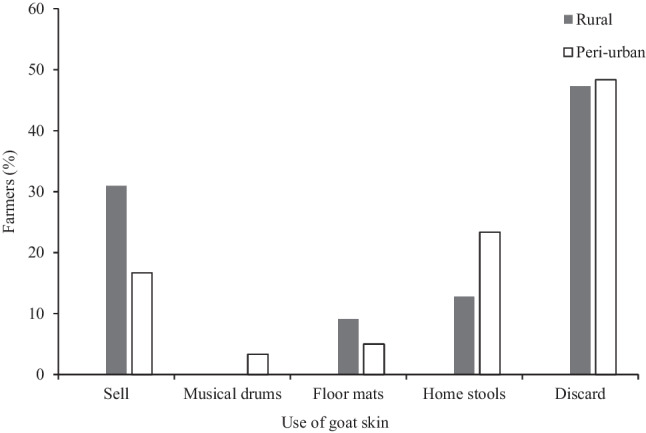
Use of goat skins in rural and peri-urban areas in KwaZulu-Natal, South Africa

Goat manure was used by 82% of respondents in rural areas and 67% in peri-urban areas. None of the farmers used goat milk. Goat skins were used for several purposes in the two sites (Fig. 2), but most (≈ 50%) were discarded. Rural areas had the highest number (31%) of farmers who sold goat skin after slaughtering an animal, while only 16% of respondents in peri-urban areas sold goat skin. Goat skins were used to make various home craft items (e.g. stools, mats) in peri-urban (23%) and in rural areas (13%). Goat skins were also used to make musical drums in peri-urban areas, but this was not mentioned at all in rural areas.

**Fig. 3:**
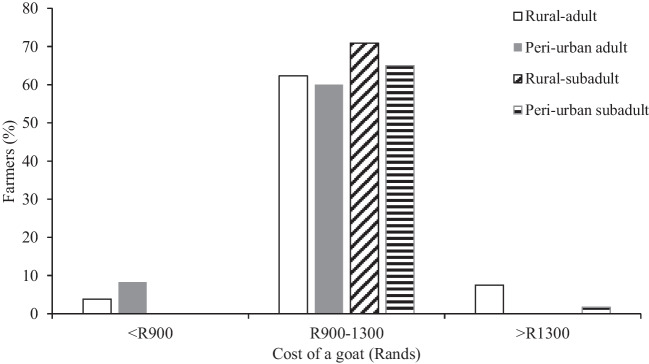
The sale income from adult and subadult goats in rural and peri-urban areas of KwaZulu-Natal, South Africa. At the time of the questionnaire survey, US$1 ≈ ZAR17.81

The sale value of an adult and subadult goat in rural and peri-urban areas was ZAR900-1300 (Fig. 3). In rural areas, adult goats could be sold for > ZAR1300, while subadults were sold for the same amount in peri-urban areas. There were rare instances where farmers in both areas would sell adult goats for less than ZAR900. The sale of goats took place mainly at homesteads (83.5%), which could be amongst neighbours or people from within the same locality or municipal area, or at formal markets or auctions (16.5%) where the seller and buyer could be unknown to each other. Goats sold at homesteads fetched lower prices than at formal markets (data not shown), but distant markets are associated with transport costs, which are exacerbated when the animals are not bought, and there is a possibility of contracting and bringing diseases to the herd. The small number of goat farmers participating in livestock auctions precluded detailed analysis of the numbers of farmers participating in near vs distant markets, and the frequency with which they participated in such markets (Fig. S2).

**Fig. 4:**
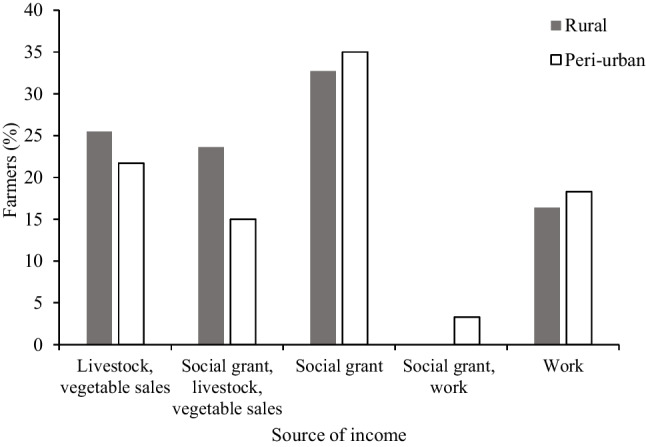
Sources of income in rural and peri-urban areas in KwaZulu-Natal, South Africa

The major contributing factors to household income were similar between rural and peri-urban areas (*χ*^2^ = 7.781, df = 6, *P* = 0.352). However, the government social grants contributed more than the income from sale of vegetables, employment, and other livestock in rural and peri-urban areas. Only 3% of respondents relied on both social grants and employment for household income in peri-urban areas; in rural areas, the combination of the two was not common amongst households (Fig. 4).

## Discussion

There were greater numbers of goats per household found in rural areas than in peri-urban areas. Spatial constraints in the peri-urban environment could explain the lower livestock numbers and smaller herds (Peacock 2005). In peri-urban areas, there may also be municipal by-laws in existence that regulate the presence of livestock within or closer to the big cities (see Grace et al. 2015; Thondhlana et al. 2022). Also, agricultural practices such as livestock husbandry in peri-urban areas were identified as a constraint by city authorities towards achieving the provision of clean water and sanitation (Ngure et al. 2019). There are also notions that animals pollute and disrupt urban spaces, and cause discomfort to urban residents (Thondhlana et al. 2022). This negative attitude towards livestock husbandry in cities may lead to the persecution and eradication of livestock from the cities and peri-urban areas. Despite this, there is a presence of small livestock in peri-urban areas, like chicken, goats and sheep, because keeping livestock is a common practice in urban areas in South Africa, and elsewhere in the world (Grace et al. 2015; Thondhlana et al. 2022). There is likely more freedom and opportunities to keep livestock in rural areas, coupled with an intact tradition, persistent capacity for animal husbandry and access to land, hence the relatively large herds found there. This might also be supported by the abundance of natural feed and natural sources of water for livestock in rural areas compared to peri-urban areas (Omiti et al. 2009; Grace et al. 2015).

The use of goat products was greater in rural areas. For example, farmers in rural areas had higher use of goat manure than peri-urban areas. This is unsurprising as crop farming takes place to a greater extent in rural areas than in peri-urban areas. Alternatively, cost and access to fertilisers may constrain rural farmers’ use more than peri-urban farmers, resulting in greater manure use in the former areas. Peri-urban areas are likely limited by space as the size of homesteads and backyards for gardens and farming is small (Galhena et al. 2013). Space allocated to corrals (where the animals are kraaled at night) and gardens in peri-urban areas would thus also be small, which may discourage crop farming (Thondhlana et al. 2022). For example, one barrier to crop farming is the potential conflict with neighbours when they own livestock that may break into fields (Thondhlana et al. 2022). In addition, the limitations in space may have likely resulted in reduced herd sizes of livestock in peri-urban areas and the amount of manure produced, for which peri-urban farmers had no use. Consequently, an increased number of animals likely results in greater production of manure.

We found that goat milk had no use to farmers in either rural or peri-urban areas. Similarly, Idamokoro et al. (2019) reported that the potential use of goat milk for consumption was low for small-scale farmers in the Eastern Cape, South Africa. This may be due to low awareness of its nutritional importance, but may also be a result of negative perceptions of associating the use of goat meat or goat products with being poor (Ngomane et al. 2022). Goat skin did not seem to have high value in either context, although some used it for cash, musical drums, floor mats and home stools. Dovie et al. (2006) reported similar uses for goat skin in Thorndale, a communal area in Limpopo, South Africa. The monetary value for goat skin for rural farmers was higher than that of peri-urban farmers. Thus, goat products appeared to have minimal household importance in peri-urban areas. The reasons for this are unclear, but may be due to the availability of alternatives, or the erosion of traditional culture in these settings.

Goats were likely more expensive in peri-urban than rural areas as we found that the cost of a subadult goat was similar (> ZAR1300) to that of an adult goat in rural areas. This may indicate different buyer intentions, which in turn influences pricing. For example, buyers in peri-urban areas may be interested in buying goats to build their herds through reproduction, and, thus, the emphasis on subadults. In contrast, buyers in rural areas may be doing so to slaughter, in which case, adults are preferred. In addition, goat prices are determined by several physical factors like colour and body condition in male goats, and the reproductive potential in female goats (Barham and Troxel 2007). The high abundance of goats in rural compared to peri-urban areas may also influence the price through supply and demand, as buyers in rural areas have various options of sellers to choose from if the price offered by one seller is not favourable to the buyer, who can then approach other sellers. High value is placed on younger females due to the reproductive potential that younger livestock possess (Mellado et al. 2006). Therefore, less value is placed on old females because they are mostly past or at the end of their reproductive window (Côté and Festa-Bianchet 2001). As a result, subadult females, which are reaching their peak reproductive potential, had the highest sales with over 65% in both areas. Furthermore, goat prices may be higher in peri-urban than in the rural areas due to the presence of buyers who buy to resell, and these buyers include commercial farmers who would participate in purchasing livestock from rural and peri-urban areas to grow their enterprises (Musemwa et al. 2008; Marandure et al. 2016). Peri-urban farmers are thus likely better positioned to make a profit and frequent sales from goats than rural farmers.

Livestock were a secondary source of income for rural and peri-urban farmers after social grants. This result is similar to that of Thornton (2008), who found that there was an explicit reliance on the welfare grants by poor households to sustain livelihoods in South Africa. The government social grants are consistent (Adekunle 2013), unlike livestock sales that may not take place despite attempts. Therefore, the lack of employment opportunities resulting from a deteriorating economic outlook in South Africa suggests that there may be a continued reliance on social grants supplemented by livestock sales by many households, especially in rural areas (Rodrik 2008, but see Thondhlana et al. 2022). The unemployed farmers received social grants for themselves and in some cases, for their children (< 18 years of age) too. A few farmers (16–18%) were gainfully employed elsewhere, and their incomes can further be supplemented by remittances from adult offspring working in major towns (Ainslie 2005). Sales of goats and, to some extent, vegetables, may contribute considerably to the income generation of rural households. The frequent and urgent sales of goats for cash may contribute towards unbudgeted household needs such as medicines (Ainslie 2005; Hoag 2018). The higher profit from goat sales may be more apparent in rural than peri-urban areas owing to the former’s use of natural resources to keep livestock and cultivate crops, unlike the more densely populated and semi-developed peri-urban areas that may require additional input costs, such as in supplementary feeds (Guendel and Richards 2002; Peacock 2005). In rural and peri-urban areas, livestock such as goats remain an alternative source of boosting household income through cash sales and other additional products that come from goats. As a result, owning resources such as assets that could be liquidated in times of financial or food vulnerability can help prevent households from food insecurity and starvation (Leete and Bania 2010; Loopstra and Tarasuk 2013).

Our finding that there is limited use of additional goat products (manure, milk, skins) is surprising. For goat manure, the abandonment of crop farming that is evident in many parts of the world (e.g. Shackleton et al. 2013; 2019) could explain its limited use. The provision of social grants in South Africa coupled with cash remittances from urban areas, declining productivity of the land, low and erratic rainfall, lower investment in agricultural extension and declining labour as the youth migrate to urban areas have also been implicated in the decline in crop production in rural areas (Manyevere et al. 2014; Shackleton et al. 2019). While goat milk is shunned (Idamokoro et al. 2019), indigenous goats are also known to produce low amounts of milk which may not be adequate for the kids, let alone for human consumption (DAFF 2019). The use of goat skin for making craft items that can be sold represents a value chain potential that can be tapped into, for most respondents indicated that the skins are discarded.

Slaughtering goats for household consumption was more frequent in rural areas than in peri-urban areas. There is increased reliance on modern foods from supermarkets in urban and peri-urban areas, which leads to the abandonment of small-scale agriculture (Grace et al. 2015). Furthermore, rural farmers slaughter more goats to provide food for household consumption (Andersson and Gabrielsson 2012), and during the main holiday periods (e.g. Christmas, Easter) when families and relatives gather together for the festivities (Nkosi and Kirsten 1993). In contrast, peri-urban farmers kept livestock for commercial reasons rather than for subsistence use, and consequently slaughtered less for household consumption. Existing regulations about the backyard slaughtering of animals in peri-urban areas may restrict the number of animals they slaughter compared to rural areas, where no such conflicts or regulations exist (Blecha 2015). Slaughtering for traditional purposes was important in rural and peri-urban areas (Thornton 2008); the meat would subsequently end up being consumed in the household or by neighbours. Surprisingly, more peri-urban farmers (17%) kept livestock solely for traditional ceremonies than in rural areas (8%).

Goats are viewed as a safety net which can be rapidly converted to cash for most farmers (Hoag, 2018), which explains why high consumption of the animals at households was not favoured (but see Mdladla et al. 2017). Yet, only 9% of farmers kept their goats for commercial reasons in both areas. Goat sales may be constrained in rural areas by distance to potential markets, so that goats are only sold during certain times of the year (Nkosi and Kirsten 1993). Otherwise, small-scale farmers would have much to gain by participating in livestock markets (Guendel and Richards 2002), particularly because they may not be knowledgeable about the opportunities available for livestock sales (Ainslie 2005). Omiti et al. (2009) showed that distance to the nearest urban areas limits access to markets for small-scale farmers in Kenya. The roads from rural to urban areas also tend to be in bad condition (Musemwa et al. 2008). Therefore, farmers in peri-urban areas have better opportunities to thrive from goat sales (e.g., opportunity and pricing) than rural farmers.

## Conclusion

Our study focused on communal goat husbandry in rural and peri-urban areas in KwaZulu-Natal where Nguni goats are farmed. The value of an animal was therefore also influenced by this, and we are uncertain of the effect of goat breed on the prices at which the animal fetches, the widespread distribution of the Nguni goat in southern Africa notwithstanding.

We sought to obtain prices at which goats were sold, focusing on adult and subadult goats in rural and peri-urban areas. Another dimension about goat pricing that was not explored is the colour of the animal, which is known to play a significant role in influencing the choices of buyers. While the questionnaire survey sought to identify all uses to which goat products are put, we were baffled by the fact that none of the respondents mentioned the bracelets made from goat skin that are pervasive amongst Zulu people. Also, our visits to households in Msinga showed cultural symbols made from parts of animals, such as the bladder, fat tissue, or horns that are prominently displayed in huts, but were not mentioned in the questionnaire interviews. Future research could be designed to interrogate the value of these artefacts to the farmers and the extent to which cultural practices influence the husbandry and marketing of goats in KwaZulu-Natal and elsewhere in southern Africa. We found that the role of goats in peri-urban areas was similar to that in rural areas. However, space availability for farming in peri-urban areas is constrained, which in turn may increase the need to supplement animal feeds and/or hire herders to manage the movement of animals.

## Supplementary information

Below is the link to the electronic supplementary material.

Supplementary file1 (DOCX 255 KB)

## Data Availability

All data are available from the corresponding author on reasonable request.
