# Supplementary material for: The utilisation of domestic goats in rural and peri-urban areas of KwaZulu-Natal, South Africa
Source: Trop Anim Health Prod. 2023 May 17;55(3):204. doi: 10.1007/s11250-023-03587-3 (PMC10191910; doi:10.1007/s11250-023-03587-3)
Supplement: Supplementary file 1 — Supplementary file1 (DOCX 255 KB) [file 11250_2023_3587_MOESM1_ESM.docx]

**Supplementary Information**

Khowa et al. The utilisation of domestic goats in rural and peri-urban areas of KwaZulu-Natal, South Africa. *Tropical Animal Health and Production.*

**Table S1** Biophysical features of the study sites in KwaZulu-Natal, South Africa. Climate and vegetation attributes are derived from Mucina and Rutherford (2006), while human population densities were derived from KwaZulu-Natal Population Statistics (2011). Levels of crop and livestock production were estimated visually by AAK

| Biophysical feature | Tugela Ferry | Kokstad | Pietermaritzburg | Howick |
| --- | --- | --- | --- | --- |
| Latitude and longitude  Altitude (m.a.s.l) | 28.7416° S, 30.4617° E  777 | 30.5096° S, 29.4063° E  1302 | 29.6006° S, 30.3794° E  596 | 29.4893° S, 30.2167° E  1 050 |
| Location | Rural | Rural | Peri-urban | Peri-urban |
| Mean annual precipitation (mm) | 682 | 747 | 897 | 861 |
| Mean minimum temperature (°C) | 6 | 9.3 | 12.9 | 10 |
| Mean maximum temperature (°C) | 25 | 19.4 | 22.2 | 20 |
| Vegetation type | Thukela Valley Bushveld | East Griqualand Grassland | Midlands Mistbelt Grassland | Southern KwaZulu-Natal Moist Grassland |
| Disturbance to vegetation | Herbivory, drought | Fire, herbivory | Fire, floods, herbivory, urban development | Fire, herbivory, floods |
| Level of small-scale crop production | High | High | Medium | Medium |
| Level of communal livestock husbandry* | High | High | Medium | Medium |
| Human population density (per km^2^) | 275 | 1 429 | 1 872 | 278 |

*At all sites, goats are free-ranging but are corralled at homesteads at night.

**Table S2** The level of education and gender of small-scale goat farmers in rural and peri-urban areas in KwaZulu-Natal, South Africa

| Level of education | | Gender | | Total (%) |
| --- | --- | --- | --- | --- |
|  |  | Male (%) | Female (%) |  |
|  | None | 8 | 21 | 29 |
|  | Primary | 17 | 11 | 28 |
|  | High School | 12 | 10 | 22 |
|  | Matric | 9 | 8 | 17 |
|  | Tertiary | 3 | 1 | 4 |
| Total | | 49 | 51 | 100 |

**Fig. S1** Numbers of livestock kept by farmers in rural and peri-urban areas of KwaZulu-Natal, South Africa

**Fig. S2** Frequency of rural and peri-urban farmers’ participation in livestock trading markets of KwaZulu-Natal, South Africa
